# A meta-analysis of temporal changes of response in the placebo arm of surgical randomized controlled trials: an update

**DOI:** 10.1186/s13063-017-2070-9

**Published:** 2017-07-12

**Authors:** Karolina A. Wartolowska, Stephen Gerry, Benjamin G. Feakins, Gary S. Collins, Jonathan Cook, Andrew Judge, Andrew J. Carr

**Affiliations:** 1 NIHR Oxford Musculoskeletal Biomedical Research Unit, Old Road, OX3 7LD Oxford, UK; 2 Botnar Institute of Musculoskeletal Sciences, Nuffield Department of Orthopaedics, Rheumatology and Musculoskeletal Sciences, University of Oxford, Old Road, OX3 7LD Oxford, UK; 3 Nuffield Department of Primary Care Health Sciences, University of Oxford, Woodstock Road, OX2 6GG Oxford, UK; 4 Centre for Statistics in Medicine, University of Oxford, Windmill Road, OX3 7LD Oxford, UK; 5 MRC Lifecourse Epidemiology Unit, University of Southampton, University Road, SO17 1BJ Southampton, UK

**Keywords:** Surgery, Placebos, Randomized controlled trials, Systematic review, Meta-analysis

## Abstract

**Background:**

Temporal changes in the placebo arm of randomized controlled trials (RCTs) have not been thoroughly investigated, despite the fact that results of RCTs depend on the comparison between arms.

**Methods:**

In this update of our earlier systematic review and meta-analysis, we set out to investigate the effect of assessment time and number of visits on the magnitude of change from baseline in the placebo arm of these trials. We used linear mixed-effects models to account for within-trial correlations.

**Results:**

Across all 47 trials the magnitude of response in the placebo arm did not change with time (β = -0.0070, 95% CI -0.024, 0.010) or visit (β = -0.033, 95% CI -0.082, 0.017) and remained significantly different from baseline for at least 12 months or seven follow-up visits. Change in the placebo arm in trials with subjective outcomes was large (β_0_ = 0.68, 95% CI 0.53, 0.82) and relatively constant across time (β = -0.0042, 95% CI -0.024, 0.016) and visit (β = -0.029, 95% CI -0.089, 0.031), whereas in trials with objective outcomes the response was smaller (β_0_ = 0.28, 95% CI 0.11, 0.46) and diminished with time (β = -0.030, 95% CI -0.050, -0.010), but not with visit (β = -0.099, 95% CI -0.30, 0.11). For trials with assessed outcomes, there was no significant effect of time (β = -0.0071, 95% CI -0.026, 0.011) or visit (β = -0.032, 95% CI -0.33, 0.26); however, these results should be interpreted with caution due to the small number of studies, and high clinical heterogeneity between studies. In trials with pain as an outcome, the improvement was significant (β_0_ = 0.91, 95% CI 0.75, 1.07), but there was no effect of time (β = -0.013, 95% CI -0.06, 0.03) or visit (β = -0.045, 95% CI -0.16, 0.069), and pain ratings remained significantly different from baseline for 12 months or seven visits.

**Conclusions:**

These results are consistent with our previous findings. In trials with subjective outcomes response in the placebo arm remains large and relatively constant for at least a year, which is interesting considering that this is an effect of a single application of an invasive procedure. The lack of effect of time and visit number on subjective outcomes raises further questions regarding whether the observed response is the result of placebo effect or the result of bias.

**Electronic supplementary material:**

The online version of this article (doi:10.1186/s13063-017-2070-9) contains supplementary material, which is available to authorized users.

## Background

There is conflicting evidence regarding the change in magnitude of placebo response in randomized controlled trials (RCTs). Several studies have demonstrated that values in the placebo arm remain different from baseline for the duration of the follow up [1–3]. However, there is no consensus as to whether placebo response does [4–6] or does not [4, 7] significantly change with time, and as to whether placebo response has a time-effect curve with a peak and carry-over effect [2, 8]. Experimental studies, specifically designed to investigate temporal changes after placebo administration, typically use a very short observation time, e.g., under an hour [9, 10], so their results provide little insight into what happens in clinical trials, in which the follow up lasts weeks or months.

In the previous paper [3], we used data from our earlier systematic review of surgical RCTs with a placebo arm [11] to determine the effect of time on the effect size of the primary outcome at the primary assessment time point. [3] We observed that the effect in the placebo arm remained significantly different from baseline throughout blinded follow up, but that the timing of the assessment did not appear to affect the magnitude of effect.

Typically, meta-analyses focus on the effect size at the primary assessment time point and treat it as a single outcome measure of treatment efficacy; however, trials often report outcomes at multiple follow-up visits. In this paper, we have assessed the temporal characteristics of changes in the placebo arm using a meta-analysis of within-and between-study changes across all outcome time points for all analyzed surgical RCTs. Our aim was to verify the findings from our earlier paper by incorporating the data from all reported follow-up visits, accounting for within-study and between-study correlations, and to investigate the effect of the outcome type and number of visits on temporal changes in the placebo arm of surgical RCTs.

## Methods

### Search strategy, eligibility and data extraction

The details of the search strategy and data extraction have been published previously [11]. In brief, we searched the databases of MEDLINE, EMBASE, CENTRAL, and ClinicalTrials.gov for placebo-controlled RCTs investigating the efficacy of a surgical intervention. The databases were searched from their inception to October 2015.

Surgical intervention was defined as “any interventional procedure that changed the anatomy and required a skin incision or the use of endoscopic techniques. Dental studies and invasive procedures used to deliver a pharmacological substance or stem cells, or that aimed to alleviate symptoms by modulation, stimulation or denervation were excluded”. Placebo was defined as “sham surgery, or an imitation procedure intended to mimic the active intervention; including the scenario where a scope was inserted and nothing was done, but patients were sedated or under general anesthesia and could not distinguish whether or not they underwent the actual surgery” [11].

We only included trials that reported continuous data and presented data in a form that made it possible to calculate an effect size. From each eligible study, we extracted the lead author’s surname, the publication year, the number and timing of each treatment and follow-up visit, the primary outcome, and the mean and standard deviation (SD) of the primary outcome in the placebo arm of each trial. Outcomes were classified as “subjective”, i.e., patient-reported and depending on the patients’ perceptions and cooperation, “assessed”, i.e., subjective ratings judged by external assessors or “objective”, i.e., measured using devices or laboratory tests and independent of patients’ or observers’ perceptions, for example, weight. As most of the literature on placebo effect is related to pain studies, we also extracted data on pain outcomes, irrespective of whether these were primary or secondary outcomes, for a secondary analysis.

Study effect sizes were quantified as within-arm standardized mean differences (SMDs) between baseline and follow-up values using the Cohen’s *d* method [12]. A pretest-posttest correlation coefficient (*r*) of 0.5 was used to calculate the standard error of each SMD [3]. Where necessary, SMDs were sign-flipped to ensure that improvement was consistently presented as a reduction in SMD.

### Model for analysis of multiple time points

In order to analyze the trajectory of change in the placebo arm, we adopted a similar method to that proposed by Ishak et al. [13], who conducted a meta-analysis of longitudinal studies reporting data at a series of fixed time points, and then used linear mixed-effects models to account for correlations, both within and between studies. We adapted Ishak’s method to conduct a meta-analysis of studies that report data at many different and irregularly spaced time points, while still accounting for correlations between data points within and between studies. All standardized data points from each individual study were included. We used a random coefficients model (also known as a random intercept and slope model) with continuous time, which allows for an arithmetic description of the relationship between the measurement of interest and time from baseline [14]. The models were based on polynomials of time by adding higher-order polynomials, first as fixed effects, then as random coefficients. Decisions on which terms to include in the final model were based on Akaike information criteria. When assessing the benefit of additional fixed and random effect terms, model parameters were estimated using maximum likelihood. Restricted maximum likelihood estimation was used when fitting the final model. Each observation was weighted according to the inverse variance of the study measures at each time point. To account for the within-study correlation of observations a first-order continuous auto-regressive (CAR1) covariance structure was used, which accounts for the irregular time periods between measurements. In the scenario where observations are equally spaced, this is identical to a first-order autoregressive (AR1) structure.

In analyzing the effect of the timing of all reported follow-up visits on SMDs within the placebo arm of surgical RCTs, the intention was to look for changes that might not have been present when only the primary assessment time point was analyzed [3]. As the type of outcome has been demonstrated to have an effect on the magnitude of response in the placebo arm, we also investigated temporal changes after subgrouping trials by outcome type [3, 15]. In this case, a separate model was fitted for each subgroup (subjective, assessed and objective). We also performed meta-analyses using visit number (instead of time from baseline) as a fixed effect to investigate the effect of repeated assessments. Finally, as most of the literature on placebo is related to placebo analgesia, we analyzed temporal changes only within trials in which pain was the outcome. For the analysis of some subgroups (assessed and objective outcomes) only a small number of studies were available, and model convergence was not satisfactory; in these cases, a simpler random-intercept-only model was fitted.

In all cases, a linear term for time was found to be sufficient for both the fixed and random parts of the model; that is, adding higher-order polynomial terms did not improve the model fit. We chose to weight each observation by its inverse variance; however, removing this weighting from the models did not change any of the conclusions. Analyses were limited to 12-month follow up and to seven follow-up visits due to the small number of trials with more visits and longer follow-up periods. All meta-analyses were performed using SAS software version 9.4 (SAS Institute Inc., Cary, NC, USA) using the “mixed” and “sgplot” procedures.

## Results

### Study selection and characteristics

Information on the search strategy, Preferred Reporting Items for Systematic Reviews and Meta-Analyses (PRISMA) flow diagram, and characteristics of the identified and included trials have been reported previously [3], with an abridged version presented in Additional file 1. A total of 47 trials, involving 1744 participants, were eligible for inclusion. The median duration of blinded follow up was 6 months, with an interquartile range (IQR) of 3 − 12 months. The median number of blinded visits was 1 (IQR 1 − 3).

### Main findings

**Fig. 1:**
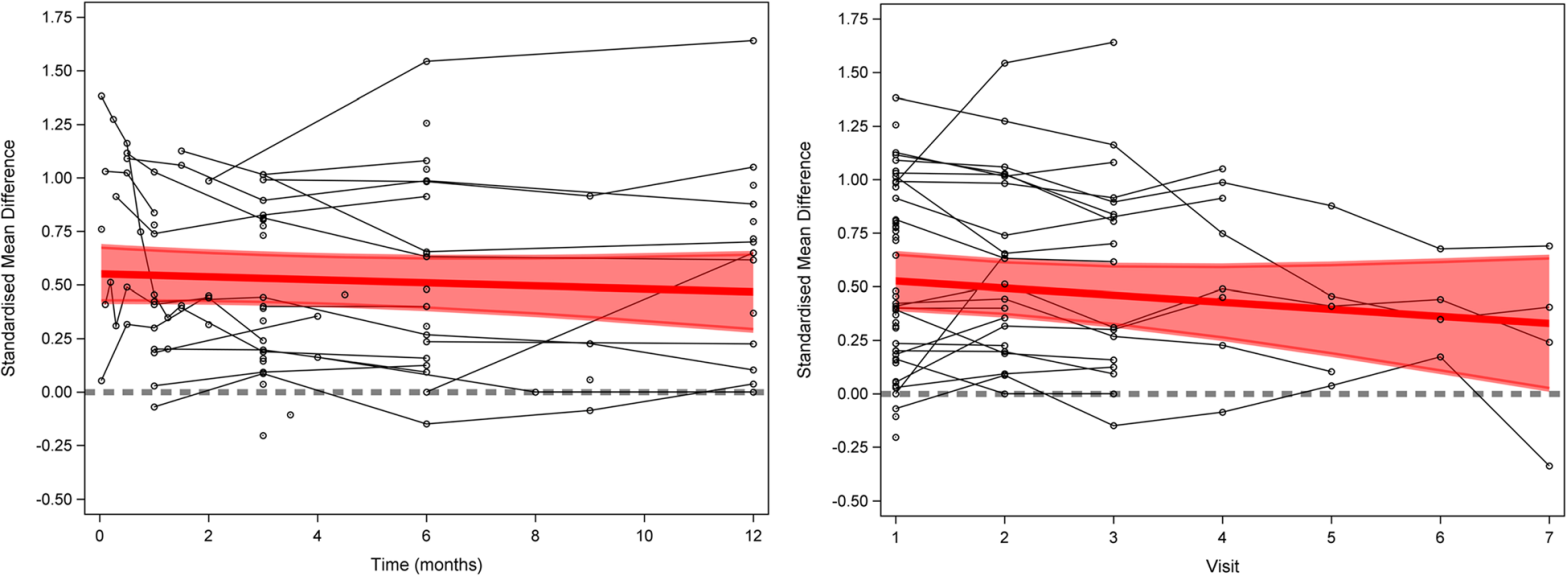
Meta-analysis of placebo response across all trials. *Left panel* shows analysis across all time points. *Right panel* shows analysis across all visits. The *thick line* refers to the study average trend over time, i.e., the fixed effects part of the model. The *shaded area* is the 95% confidence interval. The *thick grey dashed line* represents the line of null effect

Across all analyzed surgical RCTs, the magnitude of placebo response did not diminish with time or visit, and remained significantly different from baseline for the duration of the trial (Fig. 1, Tables 1 and 2).

**Table 1:** The effect of time (in months) on placebo response across all trials

Outcome type	Intercept		Coefficient	
	β_0_ (95% CI)	*P* value	β (95% CI)	*P* value
All	0.55 (0.42, 0.68)	<0.0001	-0.0070 (-0.024, 0.010)	0.40

**Table 2:** The effect of visit on placebo response across all trials

Outcome type	Intercept		Coefficient	
	β_0_ (95% CI)	*P* value	β (95% CI)	*P* value
All	0.56 (0.42, 0.71)	<0.0001	-0.033 (-0.082, 0.017)	0.16

**Fig. 2:**
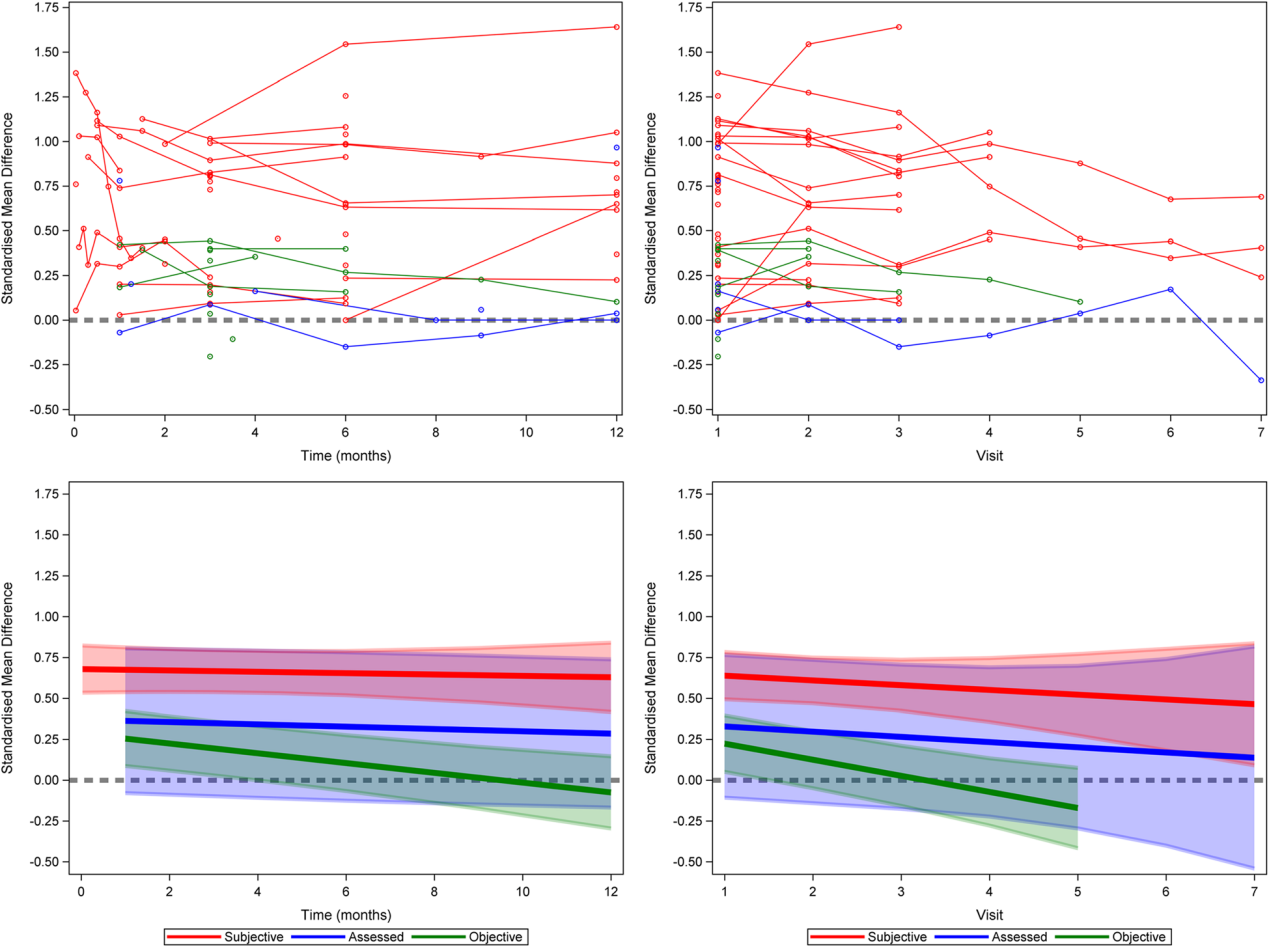
Meta-analysis of placebo response across all the trials, by outcome type. *Top panels* shows data for individual trials. *Bottom panels* shows meta-analysis by trial type. *Left panels* show analysis across all time points. *Right panels* show analysis across all visits. A separate model was fitted for each outcome type. The *thick line* refers to the study average trend over time, i.e., the fixed effect part of the models. The *shaded area* is the 95% confidence interval. The *thick grey dashed line* represents the line of null effect

When trials were subdivided by outcome type, time was associated with statistically significant decreases in placebo response in trials with objective outcomes. For trials with subjective outcomes, neither time nor visit were significantly associated with change in placebo response; however, the response remained large and significantly different from baseline for at least 12 months. Neither time nor visit were significantly associated with the magnitude of placebo response in trials with assessed outcomes, but few studies were included in this analysis (Fig. 2, Tables 3 and 4).

**Table 3:** The effect of time (in months) on placebo response across all trials, by outcome type

Outcome type	Intercept		Coefficient	
	β_0_ (95% CI)	*P* value	β (95% CI)	*P* value
Subjective	0.68 (0.53, 0.82)	<0.0001	-0.0042 (-0.024, 0.016)	0.67
Assessed	0.37 (-0.070, 0.81)	0.086	-0.0071 (-0.026, 0.011)	0.38
Objective	0.28(0.11, 0.46)	0.0037	-0.030 (-0.050, -0.010)	0.0084

**Table 4:** The effect of visit on placebo response across all trials, by outcome type

Outcome type	Intercept		Coefficient	
	β_0_ (95% CI)	*P* value	β (95% CI)	*P* value
Subjective	0.67 (0.50, 0.83)	<0.0001	-0.029 (-0.089, 0.031)	0.28
Assessed	0.36 (-0.079, 0.80)	0.091	-0.032 (-0.33, 0.26)	0.51
Objective	0.32 (0.15, 0.50)	0.0024	-0.099 (-0.30, 0.11)	0.10

**Fig. 3:**
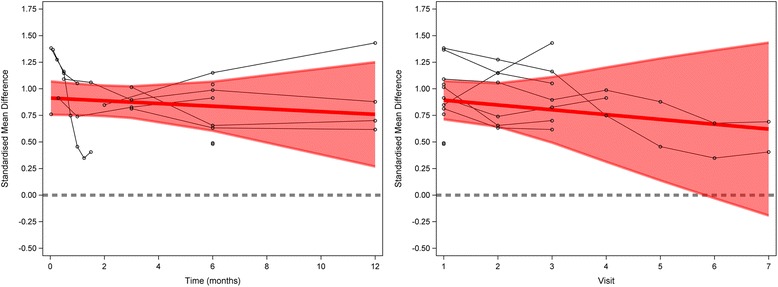
Meta-analysis of placebo response in trials with pain as the primary outcome. *Left panel* shows analysis across all time points. *Right panel* shows analysis across all visits. The *thick line* refers to the study average trend over time, i.e., the fixed effects part of the model. The *shaded area* is the 95% confidence interval. The *thick grey dashed line* represents the line of null effect

In analyses limited to trials with pain as the outcome, the effects of time and visit were not significant (Fig. 3, Tables 5 and 6).

**Table 5:** The effect of time (in months) on placebo response in trials with pain as the outcome

Outcome	Intercept		Coefficient	
	β_0_ (95% CI)	*P* value	β (95% CI)	*P* value
Pain	0.91 (0.75, 1.07)	<0.0001	-0.013 (-0.06, 0.03)	0.48

**Table 6:** The effect of visit on placebo response in trials with pain as the outcome

Outcome	Intercept		Coefficient	
	β_0_ (95% CI)	*P* value	β (95% CI)	*P* value
Pain	0.94 (0.72, 1.15)	<0.0001	-0.045 (-0.16, 0.069)	0.36

## Discussion

### Main findings

To the best of our knowledge, this is the first meta-analysis modelling longitudinal changes in the magnitude of effect in the placebo arm of surgical RCTs across all follow-up visits. This study supports our earlier findings that, across all analyzed trials, the magnitude of placebo response does not significantly change with time; it also shows that placebo response does not change with the number of visits. However, when trials were subdivided by outcome type, for trials with subjective outcomes, the response in the placebo arm remained large and relatively constant for at least 12 months or seven visits, while for trials with objective outcomes, the improvement in the placebo arm diminished with time but not with follow-up visits. It should be noted that, unlike pharmaceutical trials, in which there may be multiple applications of a placebo regimen, all but three trials analyzed in this study used a “one-off” placebo intervention (Additional file 1).

### Strengths and limitations

The main strength of this paper is that, unlike our previous analysis, we investigated the effect of time and visit number across all follow-up visits. The main limitation of this study is the paucity of data; both in the form of the number of eligible studies, and the number of observations per study.

We were not able to investigate the extent to which placebo response was caused by the so called “true placebo effect”, i.e., the difference in effect between the placebo and non-interventional arms [16], because only one trial included a non-intervention group [17]. Analyses were limited to the assessment of “placebo response”, i.e., the total change in the placebo arm between the baseline and follow-up visits. As a consequence of this, the magnitude of response may be affected by factors that are not related to placebo intervention itself, such as regression to the mean, fluctuations in disease severity, spontaneous improvement or report bias.

Seventy-nine percent of the trials used or allowed the use of standard or rescue medication [3]; therefore, some of the effect observed in the placebo group may be the result of concomitant pharmacological treatment or lifestyle modifications. We did not include any information about concomitant medication in our analysis because of the limited reporting of this characteristic in the analyzed trials.

Analyses were based on summary data, as reported by the authors of each trial. This limited the power of our analyses and did not allow us to investigate the effect of patient-related factors; therefore, we were unable to draw inference about individual patient responses (significant relationships at the population level may not hold true at the individual level and vice versa, i.e., the ecological fallacy).

The statistical modelling carried out in this study relied on several choices and assumptions. We chose to use SMDs for the outcome measure, as the reported outcome types and measures were heterogeneous, rendering standardization necessary to combine results. In a study with baseline and multiple follow-up outcome assessments, we would typically adjust for the magnitude of the outcome at baseline, but this was not appropriate here. There is also the possibility that the standard error of each study estimate was not appropriately accounted for, since the baseline value was incorporated in the SMD and the weighting at each time point; however, removing the weighting from the model made little difference to the findings.

### Interpretation

This study confirms our previous findings that placebo response does not seem to have a response-curve and that it persists for at least 12 months [3].

The “longevity” of placebo response has been reported previously [1, 2] and has been interpreted by some authors as regression to the mean [18–20]. In this study, we could not investigate whether placebo response was caused by regression to the mean because of the lack of information on the true population mean for the outcome measures. However, regression to the mean could explain many of the characteristics of placebo response, for example, that it tends to be larger for more extreme values and for more unreliable measures. [20].

Using longitudinal analysis showed that outcome type affects not only the magnitude of response in the placebo arm, but also its temporal changes; with the effect on objective outcomes decreasing with time, and the effect on subjective outcomes, including pain, not changing with time and remaining significantly different from baseline values. This is in line with the results of our original paper [3]. Findings in previously published studies, investigating temporal changes in the placebo arm have been inconsistent. Some studies reported that the effect of time is not significant [4] or that trial duration may explain some of the variation in improvement [21]. A meta-analysis of acupuncture trials [2], showed a peak in placebo response at 12 weeks with a subsequent drop at 52 weeks; however, the follow-up visits were divided into six groups according to the assessment timing, allowing a different number of trials to contribute to the effect in each group. Kaptchuk et al. [5] reported a continuous reduction in pain over 8 weeks in a sham acupuncture trial; however, they re-applied the treatment throughout the study, and it is known that administering treatment multiple times reinforces the placebo effect [22].

In our meta-analysis, visit number did not have a significant effect on the magnitude of placebo response. This contradicts the findings of Vase et al., who reported that a larger number of face-to-face visits was associated with a larger placebo response [23]. However, Vase et al. used individual patient data from several pharmacological trials, in which a placebo was administered multiple times during the trial, whereas we analyzed trial-level data from surgical trials, which tend to involve a “one-off” intervention.

Some of the improvement in the placebo arm might have been related to the effect of concomitant treatment. One plausible explanation for improvement is the use of rescue medications, such as pain-killers, or the introduction of lifestyle modifications, such as diet. Another possible explanation is that patients who were allowed to continue their standard pharmacological treatments throughout the trial might have improved due to better adherence to these treatments when in the trial.

### Implications

There are two main implications of this study. First, for the researchers and clinicians designing placebo-controlled trials of invasive procedures using subjective outcomes, it is useful to know that the effect in the placebo arm does not seem to change significantly with time. This is important because in a placebo-controlled trial treatment effect is measured as the difference between the change in the active and placebo arms. Second, patients report significant improvement for a prolonged period of time after a single surgical placebo intervention, i.e., a procedure chosen to have no active therapeutic effect. Therefore, surgeons and therapists should be aware that large and sustained improvement in subjective outcomes after a procedure does not always mean that the crucial surgical element of the treatment is effective.

## Conclusions

This study found that for surgical RCTs with subjective outcomes, the effect size in the placebo arm persists for at least a year, and does not significantly diminish with follow-up visits. Only in trials with objective outcomes did the magnitude of placebo response diminish with time, but not with number of visits. These findings further reinforce the value of controlling for potential bias, especially in trials with a subjective key outcome. Further research is needed to explore the extent to which the observed response in the placebo arm is explained by response bias and other potential sources of bias.

## Additional file

Additional file 1: Characteristics of surgical randomized controlled trials with a placebo arm included in this analysis. (PDF 47 kb)
