# Supplementary material for: A meta-analysis of temporal changes of response in the placebo arm of surgical randomized controlled trials: an update
Source: Trials. 2017 Jul 12;18:323. doi: 10.1186/s13063-017-2070-9 (PMC5508709; doi:10.1186/s13063-017-2070-9)
Supplement: Additional file 1: — Characteristics of surgical randomized controlled trials with a placebo arm included in this analysis. (PDF 47 kb) [file 13063_2017_2070_MOESM1_ESM.pdf]

	Study	Year	Country	Condition	Procedure	Placebo intervention	Standard treatment	Continuous vs. non-continuous rescue medication	Number of patients in the placebo arm	Blinding	Randomisation ratio	Number of treatment visits	Number of the primary assessment visit	Timing of the primary outcome visit [months]	cross-over	Outcome	Outcome type
1	Abbott et al.	2004	UK	Endometriosis	Laparoscopy + excision	Laparoscopy + staging	No information	not mentioned	19	Patients and assessors	1to1	single event	1	6	compulsory	Pain and QoL	subjective
2	Arts et al.	2012	Belgium	Gastroesophageal Reflux Disease	Endoscopy + RF treatment	Endoscopy + setup but no RF	Proton pump inhibitors	continuous	11	Patients and assessors	1to1	single event	1	3	compulsory	Distensibility	objective
3	Bradley et al.	2002	USA	Osteoarthritis	Tidal irrigation	Saline injection sub cut. and leg manipulation	Analgesics	rescue	91	Patients and assessors	1to1	single event	1	12	none	Pain and function	subjective
4	Buchbinder et al.	2009	Australia	Osteoporotic vertebral fracture	Vertebroplasty	Injection of anaesthetic but not cement +cephalosporin	Analgesics and standard medication for osteoporosis	continuous	40	Patients and assessors	1to1	single event	3	3	none	Pain	subjective
5	Buryk et al.	2011	USA	Ankyloglossia	Frenotomy	Sham	no information	not mentioned	28	Patients	1to1	single event	1	12 but no longer fully blinded after 1st day	optional	Pain and function	subjective
6	Castro et al.	2010	USA, Canada, Netherlands, Israel, Belgium	Severe asthma	Bronchoscopy + radiofrequency treatment	Bronchoscopy + placebo procedure	Asthma medication	continuous	98	Patients and assessors	2to1	single event	2	6	none	QoL	subjective
7	Corley et al.	2003	USA	Gastroesophageal Reflux Disease	Endoscopy + RF treatment	Endoscopy + setup but no RF delivery	Antacids for 21 days then asked to discontinue but allowed as rescue medication	encouraged to discontinue	29	Patients and assessors	1to1	single event	1	6	optional	Symptoms and QoL	subjective
8	Dowson et al.	2008	UK	Migraine	Patent foramen ovale closure with an implant	Skin incision in the groin + transesophageal US + aspirin and clopidogrel - no heparin	Prophylactic medication for 90 days (aspirin+clopidogrel)	encouraged to discontinue	73	Patients and assessors	1to1	single event	1	9	none	Pain frequency	subjective
9	Eid et al.	2014	USA	Obesity	Endoscopy + gastroplication (StomaphyX)	Endoscopy	No information	not mentioned	31	Patients	2to1	single event	5	12	none	Weight loss	objective
10	Fockens et al.	2010	USA , Netherlands	Gastroesophageal Reflux Disease	Endoscopy + Gatekeeper prosthesis	Endoscopy + saline instead of prosthesis and instead of antibiotics	PPI for 2weeks then asked to discontinue	encouraged to discontinue	43	Patients	2to1	resham at 3mo	1	6	optional	AEs and heartburn symptoms from QoL questionnaire	subjective
11	Freed et al.	2001	USA	Parkinson's disease	Cell transplantation	Incomplete trepanation (dura intact) + PET + MRI + phenytoin - sham-transplantation	Antiparkinsonian drugs	continuous	20	Patients and assessors	1to1	single event	3	12	optional	Parkinsonism (UPDRS is secondary)	assessed
12	Freeman et al.	2005	Australia	Chronic discogenic pain	Electrothermal therapy	Catheter inserted but not connected + cephazolin +CT	Rehabilitation and analgesics	continuous	19	Patients and assessors	2to1	single event	1	6	none	LBOS, SF-36, Pain and function	subjective
13	Friedman et al.	2008	USA	Sleep apnea	Palatal implant	Identical implementation device without an implant + a-biotics	Prophylactic antibiotics and analgesics	postop	31	Patients and assessors	1to1	single event	1	3	optional	AHI	objective
14	Genco et al.	2006	Italy	Obesity	Endoscopy + balloon	Endoscopy but not balloon + diet??	Omeprazole and diet	continuous	16	Patients and assessors	1to1	period of time	1	3	compulsory	Weight loss	objective
15	Gillespie et al.	2010	USA	Sleep apnea	Palatal implant	Identical implementation device without an implant	No information	not mentioned	25	Patients and assessors	1to1	single event	1	1.5	optional	CPAP pressure	objective
16	Gross et al.	2011	USA, Germany	Parkinson's disease	Cell transplantation	Scalp incisions and partial-thickness burr holes + MRI - the same duration	Levodopa	continuous	36	Patients and assessors	1to1	single event	1	12	none	Off-state motor UPDRS score	assessed
17	Guyuron et al.	2009	USA	Migraine	"Deactivation" of trigger points	Exposure of muscles and nerves without changing their integrity	No information	not mentioned	26	Patients and assessors	2to1	single event	1	12	optional	Pain frequency	subjective
18	Holmlund et al.	2014	Sweden	Snoring	Radiofrequency treatment	Setup but no energy delivery	Post-op analgesia	postop	15	Patients and assessors	1to1	up to 3 treatments at 4-6week intervals	1	12	none	ESS	subjective
19	Hurwitz et al.	2014	USA, Canada, Israel, Italy, Russia	Cancer	Sonication	Setup but no energy delivery	Analgesics	continuous	35	Patients	3to1	single event	7	3	optional	Response (pain and medication)	subjective
20	Kallmes et al.	2009	USA, UK, Australia	Osteoporotic vertebral fracture	Vertebroplasty	Simulated (audio, sensory, even smell) vertebroplasty - injection of anaesthetic but not cement	Analgesics	rescue	63	Patients and assessors	1to1	single event	3	1	optional	Disability and pain	subjective
21	Kapural et al.	2013	USA	Chronic discogenic pain	Radiofrequency treatment	No energy delivery	Back braces, analgesics, muscle relaxants	rescue	30	Patients and assessors	1to1	single event	3	6	optional	Physical function using SF-36 questionnaire	subjective
22	Koutsourelakis et al.	2008	Greece	Sleep apnea	Septoplasty	Simulated resection with manipulation of instruments - the same amount of time	No information	not mentioned	22	Patients and assessors	1to1	single event	1	3.5	optional	AHI	objective
23	Kvarstein et al.	2009	Norway	Lower back pain	Radiofrequency treatment	Sham	Analgesics	rescue	10	Patients and assessors	1to1	single event	2	12	none	Pain	subjective
24	Landorf et al.	2013	Australia	Plantar callus	Real scalpel callus debridement	Sham scalpel callus debridement	No information beyond "usual care"	not mentioned	39	Patients and assessors	1to1	single event	7	1.5	none	Pain	subjective
25	Larson et al.	1998	USA	Prostatic hyperplasia	Thermoablation	Setup but no energy delivery	Prophylactic antibiotics and analgesics	postop	42	Patients and assessors	3to1	single event	3	6	optional	AUA Score, Qmax, postvoid residual, QoL	subjective
26	Leon et al.	2005	USA	Coronary disease	Percutaneous myocardial laser revascularisation	Setup but no laser procedure	Antianginal medication	continuous	102	Patients and assessors	2to1	single event	2	12	none	Exercise duration	assessed
27	Lopes et al.	2014	Brazil	Obsessive-compulsive disorder	Gamma Ventral Capsulotomy	Gamma knife doors remained closed during procedure	Pharmacological standard treatment and cognitive behavioural therapy	continuous	8	Patients and assessors	1to1	single event	1	12	optional	Y-BOCS	subjective
28	Martinez-Brocca et al.	2007	Spain	Obesity	Endoscopy + balloon	Endoscopy	Proton pump inhibitors and diet	continuous	11	Patients and assessors	1to1	period of time	2	4	none	Weight-loss	objective
29	Maurer et al.	2012	Germany	Sleep apnea	Palatal implant	Identical implementation device without an implant	No information	not mentioned	11	Patients and assessors	1to1	single event	1	1.5	none	AHI	objective
30	McVary et al.	2014	USA, Australia	Prostatic hyperplasia	Prostatic Urinary Lift	Rigid cystoscopy	Phosphodiesterase inhibitors	continuous	66	Patients and assessors	2to1	single event	1	3	optional	PUL voiding symptoms (IPSS)	subjective
31	Moseley et al.	2002	USA	Osteoarthritis	Arthroscopy + debridement OR lavage	Skin incision without arthroscopy	Walking aids, exercise, analgesics	continuous	60	Patients and assessors	2to1	single event	7	24	none	Pain	subjective
32	Navada-Castaneda et al.	2003	Mexico	Dry eye	Lacrimal occlusion with collagen plug	Sham procedure without a plug	Artificial tears	continuous	30	Patients and assessors	1to1	single event	4	2	none	Conjunctivitis symptom score	subjective
33	Nease et al.	2004	USA	Turbinate hypertrophy	Radiofrequency treatment	Sham	Analgesics	postop	16	Patients	1to1	single event	1	6 but cross over at 8weeks	optional	VAS obstruction	subjective
34	Olanow et al.	2003	USA	Parkinson's disease	Tissue/cells transplantation	Partial burr holes + a-biotics + cyclosporine + PET	Antiparkinsonian drugs	continuous	11	Patients and assessors	2to1	single event	9	24	optional	UPDRS	assessed
35	Pauza et al.	2004	USA	Chronic discogenic pain	Intradiscal electrothermal therapy	Introducing a needle onto the disc (visual and auditory feedback)+discography + CT + prophylactic a-biotics + analgesics + rehabilitation.	Post-op analgesia, rehabilitation	continuous	27	Patients and assessors	1to1	single event	1	6	none	Pain, disability, SF-36, QoL	subjective
36	Powell et al.	2001	USA	Turbinate hypertrophy	Radiofrequency treatment	Setup but no energy delivery	Analgesics	rescue	5	Patients and assessors	3to1	single event	1	1	none	Nasal obstruction VAS	assessed
37	Rodriguez et al.	2009	Chile	Diabetes mellitus type 2	Endoscopy + bypass liner	Endoscopy + no device	Diet, metformin, sulfonylurea	continuous	6	Patients	3to1	period of time	2	6	none	HbA1	objective
38	Roehrborn et al.	2013	USA, Australia, Canada	Prostatic hyperplasia	Cystoscopy + prostatic urethral lift implant	Cystoscopy + simulated procedure	No information	not mentioned	66	Patients and assessors	2to1	single event	3	3	optional	AUASI	subjective
39	Rothstein et al.	2007	USA, Germany, Belgium	Gastroesophageal Reflux Disease	Endoscopy + plication	Endoscopy + setup but device not activated	antacids reduce and discontinue after 1 week	encouraged to discontinue	72	Patients	1to1	single event	1	3	optional	QoL	subjective
40	Schwartz et al.	2007	Netherlands	Gastroesophageal Reflux Disease	Endoscopy + EndoCinch plication	Endoscopy + setup without needle and thread loaded	Proton pump inhibitors and asked to discontinue after 1 week	encouraged to discontinue	20	Patients and assessors	1to1to1	single event	1	3	optional	Heartburn frequency	subjective
41	Sihvonen et al.	2013	Finland	Degenerative meniscus tear	Arthroscopic partial meniscectomy	Arthroscopy and sham	Analgesics and exercise	rescue	76	Patients and assessors	1to1	single event	3	12	optional	Pain and function	subjective
42	Silverberg et al.	2008	USA	Alzheimer's disease	Ventriculoperitoneal shunt	Identical shunt but occluded	Antidementia medication	continuous	88	Patients and assessors	1to1	period of time	1	9	optional	Mattis Dementia Ratings Scale	assessed
43	Siproudhis et al.	2007	France	Faecal incontinence	Elastomer implants	Saline injection	Metronidazole and paracetamol	postop	22	Patients and assessors	1to1	single event	1	3	none	Success of treatment measured with CC_FI	subjective
44	Stuck et al.	2005	Germany	Snoring	Radiofrequency treatment	Device was inserted but not activated	No medication	no meds	13	Patients and assessors	1to1	2 sessions 4-6weeks apart	1	3	none	VAS snoring and ESS	assessed
45	Swank et al.	2003	Netherlands	Abdominal pain	Laparoscopy + adhesiolysis	Laparoscopy	Analgesics	rescue	48	Patients and assessors	1to1	single event	3	12	optional	Pain and QoL (pain)	subjective
46	Thompson et al.	2013	USA	Obesity	Endoscopy + outlet reduction	Sham	Diet	continuous	27	Patients and assessors	1to1	single event	3	6	none	Weight loss	objective
47	Wood et al.	2014	USA	Emphysema	Bronchoscopy + valve	Bronchoscopy + no valve	Medical management	continuous	135	Patients and assessors	1to1	single event	3	6	none	QoL	subjective
